# Blockchain technology diffusion in tourism: Evidence from early enterprise adopters and innovators

**DOI:** 10.1016/j.heliyon.2024.e24675

**Published:** 2024-01-14

**Authors:** Yin Maythu, Andrei O.J. Kwok, Pei-Lee Teh

**Affiliations:** a Faculty of Tourism, University of Girona, Spain; b Faculty of Humanities, University of Southern Denmark, Denmark; c School of Economics and Business, University of Ljubljana, Slovenia; d Department of Management, School of Business, Monash University Malaysia, Malaysia

**Keywords:** Competitive advantage, Diffusion of innovation, Blockchain, Research agenda, Motivators and drivers, Challenges and barriers

## Abstract

The use cases of blockchain as an innovative technology have increasingly captured the attention of tourism enterprises. To date, the literature tends to discuss blockchain's advantages rather than how early enterprise adopters and innovators experience and perceive the technology. As such, the extent of technology diffusion is not well understood. This study critically explores the factors influencing blockchain diffusion in tourism and how blockchain innovation is diffused in tourism. We conducted semistructured interviews with founders and senior executives of tourism enterprises in the United States and Europe who are early adopters and innovators of blockchain in tourism. From the thematic analysis, our empirical findings indicate that blockchain has much to offer despite the nascent link between blockchain's business value to an enterprise's strategic plans and the limited success of use cases in tourism. We summarize the findings in a conceptual framework and offer propositions based on the antecedents (motivators and drivers and challenges and barriers) of blockchain diffusion of innovation for enterprises to achieve competitive advantage. The propositions provide a research agenda to guide the strategic implementation of blockchain.

## 1 Introduction

In a competitive landscape, tourism enterprises need to thrive to win market share and develop a unique advantage. Tourism enterprises can achieve a competitive advantage via cost-effectiveness or product or service differentiation. Since tourism products are more or less similar, a well-developed revenue management strategy can play a vital role. Therefore, tourism service providers need to find ways to lower distribution costs to maximize their profit [1]. Lowering distribution costs is not possible when there are many intermediaries in the tourism value chain [2,3].

Intermediaries such as online travel agents (OTAs) and global distribution channels (GDCs) tend to dominate the present tourism ecosystem and force high distribution costs on small and medium tourism enterprises that rely on them to stay competitive [4]. Although these businesses have seen and experienced the monopoly acts of intermediaries and are dissatisfied and have conflicts with them [5], their competitiveness is contingent on the chosen intermediaries rather than solely on the quality of service that these tourism enterprises provide [3]. Given this challenge, blockchain technology offers tourism enterprises an innovative opportunity to attain a competitive advantage by differentiating themselves and reducing costs.

Blockchain technology is a peer-to-peer database composed of a chain of blocks where peers authenticate transactions in the blockchain network [6], eliminating the need for a middleman to verify the records. Since transactions on the blockchain are transparent and immutable, the data behind the transactions are secure and safe from manipulation, thus imbuing the blockchain with the inherent qualities of efficiency, trustworthiness, and security. For tourism, the blockchain creates real-time data flow for reservation and distribution systems by removing intermediaries. For example, blockchain-driven cryptocurrencies eliminate intermediaries for transactions such as banks [7]. As a result, tourism enterprises can process transactions faster and from reliable data sources [8]. The use of blockchain also fosters efficiency, cheaper distribution costs, secure and transparent transactions, and guest satisfaction and elevates trustworthiness [9].

Blockchain-based applications can replace traditional loyalty programs, resolve rate integrity issues, and manage service providers’ credentials and traveler identity [10]. Early adopters of blockchain technology have seen improvements in their competitive advantage and brand positioning [11]. For instance, Winding Tree connects service providers and clients without the need for intermediaries [12]. Another example is Nordic Choice Hotels, which successfully adopted blockchain technology to reduce their distribution costs while offering more competitive prices and value-added services to their guests [13].

Technically, there are various blockchain systems based on “different data structures and consensus mechanisms” [14]. Basically, the three types are public, private and permissioned. A public blockchain (e.g., Bitcoin, Ethereum) is an open system whereby anyone can freely participate, whereas a private blockchain is a closed system that limits participation to authorized parties. Finally, multiple enterprises govern a permissioned or consortium blockchain, which functions as a semiclosed system (public‒private hybrid) [14,15]. Therefore, depending on the blockchain system used and the attributes, application areas differ between consumer-to-consumer (or peer-to-peer) and consumer-to-business cases, such as cryptocurrency payments [16]. Applications in business-to-business from pure cooperation [17] can turn into coopetition via shared loyalty programs [18].

Blockchain's diffusion of innovation in tourism is still limited despite the potential benefits mentioned. While existing studies have examined the perspectives of individual tourists [19,20], the business implications for tourism enterprises have not been sufficiently examined. In addition to Chen and Tham's [21] specific investigation of merchants' receptivity of cryptocurrency adoption for tourism activities, Toufaily et al. [22] discussed blockchain's diffusion of innovation at the macro (sectoral) levels. However, empirical studies have been lacking, especially from the perspective of innovators and adopters and from experiences at the micro (organizational) levels. To address the research gap, this study sets out to explore blockchain adoption behavior, the factors influencing blockchain technology diffusion in tourism enterprises and how its innovation diffuses in tourism. To do this, we ask the following questions:

1. What factors influence early enterprise adopters and innovators' blockchain adoption in tourism leading to its diffusion of innovation? Specifically, we aim to examine the antecedents in terms of the (i) drivers and motivators and (ii) challenges and barriers.
2. How has blockchain innovation diffused in tourism? Specifically, we aim to investigate the factors of blockchain's diffusion of innovation in the tourism ecosystem.

Drawing from the empirical findings, this study develops a conceptual framework and offers propositions for a research agenda to guide the strategic implementation of blockchain toward achieving competitive advantage.

## 2 Literature review

### 2.1 Theoretical background

According to Rogers [23], innovation is diffused by individuals, society, or businesses over time. Diffusion is a dissemination process that includes adopting innovation by new users, especially among small enterprises [24]. Five attributes, relative advantage, compatibility, complexity, trialability, and observability, affect a technology's broader diffusion [25]. Relative advantage refers to the perception by potential users that new technology is better than existing technology and that the implementation of such technology will provide an organization with benefits. Compatibility refers to a new technology's fitness level with existing systems, procedures, and organizational goals. Complexity considers how difficult it is for users to learn and understand. Trialability refers to the extent to which users experiment with the new technology before adoption. Finally, observability considers how easily potential users can find information about new technology. When these five attributes are favorable from a potential user's perspective, such users are more likely to adopt the new technology, and the diffusion rate is expected to be faster [25].

### 2.2 Background of blockchain adoption in the tourism sector

Blockchain diffusion is still limited to early adopters and innovators in the tourism industry. Despite the benefits and competitive advantage that blockchain technology can potentially bring to tourism businesses, the tourism sector seems far behind others in adopting this innovation [26], as enterprises face challenges and obstacles in doing so. Roger's diffusion of innovation theory is highly relevant to studying the blockchain diffusion phenomenon in tourism because it explains why and how innovations are adopted and at what rate they proliferate in various business sectors [27]. The five attributes of the diffusion of innovation theory can provide conceptual guidance to explore the factors influencing blockchain diffusion and how blockchain contributes to tourism enterprises.

The adoption and implementation of blockchain require considerable financial and supporting resources (e.g., technical assets, expertise, time) [28]. Given the infancy of the existing market structure, many enterprises are concerned about an increase in cost due to legal requirements, even though blockchain can be cost-effective [29]. While blockchain provides a secure and decentralized environment for recording and storing disintermediated transactions, its overall efficiency is limited to current business needs. It provides a small number of transactions per block and consumes a large amount of power in some applications [30]. On the flip side of the immutability and transparency of blockchain networks, users have privacy concerns [31]. Moreover, scalability and communication failures challenge blockchain diffusion [32]. For Proof-of-Work blockchains, energy consumption required for mining is a major concern [33].

The following literature review (Table 1) summarizes selected recent studies from 2020 to 2023. It serves as a valuable reference point to understand the current state of knowledge and trends, offering a foundation for further analysis and exploration.

**Table 1:** Selected blockchain studies published between 2020 and 2023.

Area	Summary	Research method	Author
Blockchain acceptance	This study uses UTAUT model to investigate the factors that determine blockchain acceptance and how social media as a learning tool influences user awareness and interest in blockchain.	Survey	Chang et al. [34]
Blockchain in tourism businesses	An empirical study on tourism businesses in China which explores blockchain's enhancement of business efficiency, transparency and cost reduction. It demonstrated blockchain's effectiveness for tourism business models and presented country-specific insights.	Econometrics	Cheng [35]
Blockchain in the hotel food supply chain	Utilizing a mixed-method approach, the study explores the importance of traceability in the food industry when considering blockchain adoption. Findings show that blockchain increases consumer trust and influences attitudes and behaviors, while supplier concerns revolve around intraorganizational support and data sharing.	Experiment and interview	Cozzio et al. [36]
Blockchain use in tourism	The study evaluates the risks of adopting blockchain technology in tourism, which consist of societal, technical, financial and legal risks. The study concludes that most of the risks are not critical and can be mitigated.	Review and Delphi	Dadkhah et al. [37]
Blockchain application in tourism	This study explores the potential applications of blockchain in tourism, including its advantages and challenges in implementation. Through topic modeling, it reviews the trends of eco-friendly blockchain, metaverse, NFTs, smart contracts, and crypto-tourism for future research.	Review	Puri et al. [38]
Cryptocurrency payments adoption in travel	In the context of South Korea and China, the study sheds light on cryptocurrency payments adoption and how adoption differs by age and gender.	Survey	Radic et al. [39]
Blockchain adoption enablers in the tourism and hospitality sector	This study analyses the enablers of blockchain adoption from the perspective of tourism and hospitality sector employees. The study finds that the priorities of drivers and barriers differ between India as a developing country and the Netherlands as a developed country.	Interview	Sharma et al. [40]
Blockchain-driven hotel reservation system and traveler type	The study explores the acceptance of blockchain-driven hotel reservation systems and traveler behavior types. Through the Agentic Theory of Human Behavior, the findings include that travelers with collective mindsets are more likely to adopt blockchain-enabled applications and that individualistic mindsets are the opposite.	Survey	Strebinger et al. [19]
Merchants' cryptocurrency adoption for tourism	A case study that investigates factors that determine merchants' receptivity of cryptocurrency adoption for tourism activities.	Interview	Tham and Chen [21]
Cryptocurrency adoption in travel and tourism	The study examines travellers' cryptocurrency usage experience for travel-related consumption. The study also reveals the positive and negative perceptual antecedents of cryptocurrency and blockchain technological contingency factors.	Survey and content analysis	Treiblmaier et al. [20]
Blockchain-driven loyalty programs	A survey study on consumers' attitudes toward traditional and blockchain-driven loyalty programs discovers the latter is preferred. The study calls for future research to understand how blockchain-based loyalty programs can benefit companies.	Twitter sentiment analysis and survey	Treiblmaier and Petrozhitskaya [18]

## 3 Methodology

### 3.1 Data collection

This study takes a company perspective by specifically identifying tourism enterprises that are early innovators and adopters of blockchain technology. Given that few tourism companies have incorporated blockchain in their operations and beyond cryptocurrencies and speculative aspects, we recruited ten founders and senior executives (e.g., CEOs and division heads) to participate in semistructured interviews regarding the implementation of blockchain by their organizations. The respondents were located in Europe and the United States, and their firms varied in size, from small and medium enterprises to large multinational enterprises of up to more than 10,000 employees. They consisted of OTAs and accommodation reservation platforms (*n* = 4), blockchain service providers (*n* = 4), and transportation sector companies (*n* = 2) (Table 2). Respondents were recruited using a combination of purposive and snowball sampling methods. The purposive sampling method was applied first to identify the companies through the internet, literature and media and through professional networks. After an interview with a participant, a snowball sampling method is applied to obtain a referral from the respondents to access other respondents. Snowball sampling is used when individuals are not easily accessible or will not respond to online advertisements (e.g., online ads call for research participants). This combination of research methods has been widely used in recent qualitative studies on blockchain and smartphone artificial intelligence (AI) functions [41,42]. Therefore, it is suitable to combine two sampling methods for study in a niche area. Ethics clearance was provided by the University of Girona, Spain, and the research was conducted in compliance with the ethical principles of the National and European Union regulations. Informed consent was obtained from all participants for the study, and the data were handled according to the European Union General Data Protection Regulation (GDPR) guidelines.

**Table 2:** Respondent profile.

Respondent	Position	Location	Type/Sector
1	Founder	United Kingdom	Blockchain Service Provider
2	Senior Executive	United States	
3	Senior Executive	Spain	
4	Founder	Estonia	
5	Senior Executive	Sweden	Accommodation
6	Founder	United States	
7	Senior Executive	United Kingdom	
8	Founder	United States	
9	Senior Executive	United States	Transportation
10	Senior Executive	France	

Each semistructured interview was conducted from 45 to 60 min online between March and May 2021 amidst restrictions on movement and economic activities during the COVID-19 pandemic. We developed the questions based on the diffusion of innovation theory (Table 3). Prior to conducting actual interviews, we conducted pretest interviews with fellow tourism researchers to refine the questions.

**Table 3:** Interview questions relevant to assessment areas.

Assessment	Semistructured interview questions
Background	1. What was the underlying reason for adopting blockchain at your company?
Relative advantage	2. What were the expected benefits of blockchain? 3. What kind of advantages and disadvantages did you have upon implementing blockchain? 4. What were the costs and advantages of this technology?
Compatibility and complexity	5. How compatible was blockchain technology with the existing systems? 6. From your organization's perspective, how complex is this technology to learn and understand?
Observability	7. Prior to adopting blockchain, how and where did you get information? 8. Why did you decide to use blockchain among all the different technology?
Trialability	9. Were you able to test prior to fully implementing blockchain?
Competitive advantage	10. How does adopting blockchain technology contribute to your business' competitiveness? 11. What are the challenges and barriers preventing blockchain adoption in tourism? 12. Would you recommend others to adopt blockchain technology? Why?

Each semistructured interview was transcribed using Descript 14.0.0 and Otter.ai software programs, and the transcripts were proofread and reviewed carefully to eliminate machine errors before being input into the NVivo version 12 software program.

### 3.2 Data analysis

Braun and Clarke's [43] six-phase theory-driven thematic model was used to analyze the interview data based on a theory-driven thematic analysis, consistent with the approach used in existing studies on the diffusion of technology [44,45]. The phases were described as follows:

• Phase 1—involved familiarizing the data in all aspects before the coding process, including reading and rereading the transcripts.
• Phase 2—the initial coding process, whereby a top-down coding approach was used to confirm overarching ideas based on the diffusion of innovation theory.
• Phase 3—involved the ‘back translation’ of the data, where the initial codes were reflected with theories to improve the reliability and validity of the linguistic data [44].
• Phase 4—the determined themes were reviewed again, filtered as necessary, and checked to verify how they answered the main research question.
• Phase 5—the themes were defined and labeled. The relationship between parent themes and subthemes was clarified.
• Phase 6—The findings are discussed below.

In thematic analysis, frequency is not the only determining factor for theme development. Patterning across collected data is necessary, yet addressing the research question is more important [46]. The main characteristic of a theme is defined by its significance [44]. How many times a text or a phrase needs to be mentioned to be called a theme is questionable with no universal agreement on it. However, in qualitative analysis, it is a decision based on researchers’ epistemological considerations [43]. The initial coding resulted in hundreds of themes or keywords that were later clustered with similar concepts. To ensure the rigor of the coding process, the top-down coding approach and line-by-line coding approach were used. Detailed coding phases are illustrated in the Appendix.

## 4 Discussion of findings

This section discusses the findings from the three thematic areas, namely, 1) the motivators and drivers, 2) the challenges and barriers, and 3) the diffusion of blockchain.

### 4.1 Motivators and drivers of blockchain diffusion

#### 4.1.1 Benefits to early adoption

The ability to access market segments earlier is an early mover advantage. The findings confirm that early adopters were motivated to be leaders in innovation in the business, similar to Toufaily et al.’s [22] study. By implementing blockchain in business operations, enterprises are able to offer new products to their customers, as supported by Guo et al. [47]. Additionally, blockchain creates an ecosystem where enterprises can easily access new suppliers that they were previously unable to reach. The respondents mentioned that this helped them create cheaper and more competitive products than those of their competitors. They claimed they could create new tourism products since they had more access to suppliers. The respondents perceived that blockchain enhanced their competitive advantage through product differentiation and publicity. At the current stage of their businesses, a monetary return on investment was still limited. For early adopters, nonfinancial intrinsic values such as prestige could be strong drivers. For example:

*“With blockchain, we could offer new services that no one has seen before because we saw that new startups or companies would be able to make packages or offer that. But at the moment, we haven't seen more than publicity and being seen as innovative.” (Respondent 5)*

#### 4.1.2 Cryptocurrency use

Blockchain and cryptocurrencies are undeniably interconnected. This is because blockchain is the underlying technology of cryptocurrencies, and Bitcoin initiated the use of blockchain as a payment system, thus making it popular [48]. Three respondents explicitly stated that cryptocurrency transactions were their underlying reasons for implementing blockchain in their business operations. The results are in line with Chen & Tham's [21] finding that cryptocurrency's novelty drives merchants' adoption. Similarly, platforms and enterprises (e.g., Travala and Locktrip) that accept cryptocurrencies also operate in their own cryptocurrencies [49,50]. Our respondents claimed, “crypto is truly the driving force behind the technology itself” and might lead to blockchain technology acceptance. For example:

*“The more Bitcoin becomes valuable, the more people look at blockchain. And the more people look at blockchain, well, here we are.” (Respondent 4)*

#### 4.1.3 Cost reduction

A common challenge in tourism businesses is the high distribution costs paid to intermediaries, especially OTAs. Having several intermediaries to reach a client or to make a sale adds to the distribution cost. Intermediaries mentioned by the respondents are OTAs, payment providers (i.e., banks), GDS, travel agencies and travel management companies. For example:

*“We have big challenges with them (OTAs) taking quite a big part of the income for the reservations. We just wanted to see if there are any other possibilities around that could do this.” (Respondent 5)*

Our findings confirm Irannezhad and Mahadevan's [51] case study on service providers such as Travelport, TUI Group, Webjet and Winding Tree. Four respondents claimed that blockchain reduces their cost of operations and products during the reservation of hotels, transportation, and flight tickets. In particular, some respondents highlighted accelerated internal procedure execution after blockchain implementation, as blockchain can reduce manual work and accelerate the customer service process. The transactions on blockchain using cryptocurrencies incurred zero overhead costs in contrast to credit card charges [21]. For example:

*“In travel industry particularly, we see at least 30% of administrative costs will be reduced, the rescheduling fees to negligible amount and they will not be needed a customer support if you ever have to cancel and reschedule. You can simply click a button. Overall efficiency improvement is 30% in our business model (due to blockchain).” (Respondent 8)*

#### 4.1.4 Data ownership

Trust is required at every level in tourism. Companies need intermediaries, as ‘trust’ is required to ensure the accuracy of transactions and the validity of the products and services offered. However, this may lead to overdependence on intermediaries [52]. Tourism enterprises tend to rely on intermediaries to sell their products and services, which puts their online reputations at risk. For instance, a hotel needs to have good reviews on websites such as Facebook, Airbnb, or Google to be seen as ‘credible’ for its quality of service. Since reviews may be of products or services offered by the intermediary on behalf of the hotel and the reviews are centralized on platforms that tourism operators do not own, negative customer reviews can disrupt or severely damage a long-established online presence overnight. The challenge, therefore, is not just about the cost of product/service distribution through intermediaries but one of self-sovereignty and data ownership [53]. There is a need for a mechanism where travelers can provide reviews directly to the hotel where they stayed or the airline they flew with. Peers can authenticate the accuracy of stays and reviews. This is a critical challenge of ‘own your data,’ and it can be resolved with the help of blockchain. This is linked to other discussions about digital self-sovereign identity. Respondents 4 and 5 noted that substituting intermediaries with blockchain technology gives hotels and travelers complete control over their reviews, data, and accounts. Respondent 3 developed a decentralized app (dApp) that links travelers to their digital identity and records users' accurate travel history.

*“We developed a digital blockchain health certificate*^1^*when nobody started. We thought that we can use that because in a way we can avoid the fake certificates. Because nobody can create a fake certificate if you are dealing with blockchain. With a digital blockchain health certificate, hospitals, health centers, and the labs, they upload information directly in the blockchain. Nobody can manipulate the documents.” (Respondent 3)*

#### 4.1.5 Business opportunities

Blockchain-driven loyalty programs are suitable for a group of companies that would like to use the same loyalty program and where traditional systems are not capable of allocating transactions between multiple companies [54]. According to Treiblmaier and Petrozhitskaya's [18] empirical study, consumers prefer blockchain-based loyalty programs over traditional programs. For example, Respondent 3, who was developing their company's loyalty program on a blockchain network, explained as follows:

*“You have only one code for one person for the consumer, for the client. And so, I can interact with all the different companies in the same loyalty program. So, there's no complications when you are trying to take or to use your points or to do any business through the loyalty program.” (Respondent 3)*

Blockchain allows disintermediation in reservation and distribution systems, providing a database for secure information storage, generating cost reductions and operational efficiency, documenting loyalty program transactions, and identity management, as posited in existing studies [9,19]. For example:

*“With blockchain, we can create a marketplace where like people can exchange rewards points. So, let's just say I had fifty thousand Hilton points. Maybe I could exchange with someone who has, you know, fifty thousand tons of miles and then make that exchange with them on their marketplace.” (Respondent 2)*

Fifty percent (50 %) of respondents pointed out how blockchain contributed to data transparency and engendered trustworthiness during their organization's technology implementation. This finding confirms Al-Breiki et al.’s [55] analysis of blockchain architecture that enables trust. For example, the firm of one of the respondents was using blockchain to establish smart contracts and document the results from performance-based agreements between hotels and corporate travelers.

*“Using smart contracts to allow companies and hotels to have an agreement on rates that is truly dynamic. It could be performance-based. So, you can have an agreement where the more the company books at that particular hotel property, the lower rate, they get the better the deal.” (Respondent 6)*

### 4.2 Challenges and barriers to the diffusion of blockchain in the tourism industry

#### 4.2.1 Technology maturity and scalability

The primary challenge to the diffusion of blockchain in the tourism industry is its scalability for current business needs. Although conceptualized in 2008, its implementation by businesses is still nascent, and tourism enterprises have yet to adopt the technology broadly. Despite the feasibility for cost reduction and disintermediation, blockchain is not yet at a scalable stage to supplant existing systems. Due to the heterogeneous characteristics of the internet, blockchain tools and services must be designed to be highly interoperable [53]. Lack of interoperability is a drawback to facilitating the scalability of the blockchain. Ferdous et al. [53] stated that self-sovereign identities and blockchain need to be compatible with legacy systems for some time to ensure collaboration between new and existing systems. The respondents' perspectives generally aligned with Muzzy and Anderson's [56] report, which suggested that blockchain networks are at risk of ‘Balkanization’ if they become isolated outlier networks. For example:

*“People do things because if they have a problem and they want the solution or do things because they are easier. And blockchain right now doesn't cover any of these two. The technology itself, it's still immature.” (Respondent 4)*

#### 4.2.2 Lack of skilled workers

According to Ge et al.’s [57] empirical study, there is a gap in fulfilling the demand for qualified and skilled blockchain workers. In our study, three respondents reported that the technology is difficult to understand and a lack of skilled workers in this field (Respondent 3, 7, and 8). Companies that develop blockchain technology internally find recruiting skilled employees a major challenge.

*“The main barrier I see is the lack of technicians that can code on the blockchain. Feel we need more people. Who knows how to program on blockchain and do it in a very... do it efficiently.” (Respondent 3)*

However, a lack of skilled workers does not necessarily imply that companies should develop blockchain technology internally. Businesses that use the technology need to understand concepts such as private and public keys; hence, they require user input more than other technologies.

#### 4.2.3 The need for user input

Blockchain demands considerable user input, and users need to understand basic concepts such as private keys, public keys, and how to manage their digital wallets [58]. Enterprises switching from Web 2.0 to Web 3.0 will drastically change their work procedures. As user input is necessary for consumer-to-business, enterprises that adopt blockchain need to know the type of blockchain that can cater to their organizational needs. Choosing the wrong type of blockchain can adversely affect customer relations and finance. Tourism enterprises must possess preexisting technology and resources [29]. Otherwise, they face functional and economic risks if they are unable to resolve the digitalization challenge. Thus, for now, blockchain diffusion is constrained to tech-savvy early adopters who are willing to take risks. This finding is identical to the results of previous studies [10,32], which found that customer readiness is a challenge.

*“In Web 3.0, you have your own account. Based on a wallet, but you have one account and to use a new system, you basically click a button, and your account connects to the new system seamlessly.” (Respondent 4)*

#### 4.2.4 Change management issues

As a challenge to blockchain diffusion, change management encompasses internal change and change at an industry level [59]. From an internal change perspective, enterprises are hesitant to make organizational changes unless their current operations face a critical issue [60]. Existing systems and tools are still serving their intended purpose in the tourism sector, so enterprises need more incentives to switch. It should be noted that blockchain changes the hierarchy within companies because it creates a more transparent ecosystem and faster information flows within a company.

Our findings support those of previous studies by Kim and Kankanhalli [61] and Walsh et al. [62], who posited that change management directly impacts technology adoption by businesses. Internal employees and executives will resist accepting new technology if the old technology still works. A respondent who commented on change management issues at the industrial level stated that a challenge to adoption could be that corporations collectively do not wish to change:

*“People don’t like change. They (blockchain technology) are a disruptor. They disrupt the whole GDS and current stack. Incentivize to not change.” (Respondent 9)*

#### 4.2.5 Monopolies as roadblocks

According to Makridakis and Christodoulou [63], blockchain has the potential to counter monopolies via decentralization and elimination of intermediaries. Seven respondents identified monopolies as potential roadblocks to blockchain adoption. It is the second largest subtheme as a challenge after scalability in terms of frequency and number of responses. Decentralization within tourism, in turn, can make major players vulnerable if they are benefiting from the current ecosystem. In the case of a completely decentralized travel marketplace, platforms that connect travelers and suppliers will have less power to intervene. When travelers can reach out directly to end suppliers, intermediaries are unnecessary. In this aspect, a decentralized travel ecosystem will positively transform the current ecosystem, making it less hierarchical and removing the need for some entities.

*“Airlines make it difficult; hotels make it difficult that the travel agencies put a lot of roadblocks. The travel agencies have a lot of leverage with the airlines. So, it makes it very difficult to change the way it’s done now.” (Respondent 9)*

Therefore, we have seen more use cases of private blockchains by major players. This leads to closed, siloed networks that are not interoperable with each other. There is a significant barrier to entry for new business models with complete blockchain implementation. While major intermediaries in the tourism sector do not have the power to hinder blockchain adoption among enterprises, they can accelerate the diffusion rate (Respondent 4) by enhancing the awareness of the technology.

#### 4.2.6 Lack of perceived benefits

Although blockchain has gained vast media attention in the tourism industry, it is challenged by the observability and visibility of actual blockchain tourism use cases. In recent years, we have seen a growing number of research articles on blockchain. However, the results prove that the economic benefits of blockchain in the tourism context are still not visible or at least in statistical evidence. Our findings build on the previous analysis by Yli-Huumo et al. [64], who stated that less than 20 % of blockchain research focuses on actual business challenges and use cases, and the rest are theoretical and technical studies.

*“The industry is too much focused-on cryptocurrency overall rather than focusing on the on the use cases of the blockchain.” (Respondent 8)*

Indeed, there is an overemphasis on the technology rather than the true underlying value to businesses [65]. Most potential users adopt new technology because it meets their needs, not how it is built. Therefore, the lack of awareness about blockchain and its business value can be a barrier to broader adoption.

#### 4.2.7 Environmental impact

The literature has identified the paradox of blockchain and cryptocurrencies [66]. Twenty percent of the respondents mentioned that the misconception of Bitcoin's synonymity with high energy consumption arising from media reporting has an adverse impact on blockchain's image and can deter potential users. Respondent 5 mentioned that *“when we started, the Bitcoin had predicted bad reputation. And it was very much linked to blockchain and Bitcoins at that time. So, we had some hard times trying to really get away from the Bitcoin world.”* Likewise, Respondent 3 claimed that *“there are a lot of misconceptions about the blockchain showing the energy inefficient.”*

While bitcoin transactions and mining tend to consume energy in public networks, using blockchain does not make an enterprise unsustainable, as it is contingent on the type and consensus mechanism used, which could be minimal for private networks. Furthermore, Ethereum, as a public network, managed to decrease its energy consumption by 99.988 % and carbon footprint by 99.992 % after switching from Proof-of-Work to Proof-of-Stake [33]. By reducing transaction inefficiencies and increasing access to the economy, blockchain contributes to economic sustainability, in line with the UN Sustainable Development Goal No. 12, which is the responsible consumption of resources and energy [67]. Respondent 3's opinion below builds on the existing call from Treiblmaier and Beck [29] call that for blockchain to diffuse wider use, it needs to ‘sustainify’. It is not only about green energy input to function but also a reconceptualization of successful operation of the blockchain networks.

*“Blockchain is quite energy-consuming and, so, if you feed your blockchain network with a renewable or green energy thing, it's okay. But if you're burning petrol to get energy to feed your blockchain network, then it is bad. But you cannot do it on your own, you need others' collaboration. And the second thing is you have to be careful about the energy because if you use a big blockchain network, you should try using green energy.” (Respondent 3)*

With the trend toward sustainable tourism, tourism enterprises are trying their best to be seen as green and energy-efficient [68]. According to the authors, sustainability is part of a strategic plan, and the paradox of unsustainable blockchain might contradict many tourism enterprises' organizational values. Media coverage of cryptocurrency mining and high energy consumption is a more concerning trend. This is the actual challenge for blockchain diffusion beyond its environmental impact. However, the environmental aspect should not be neglected.

### 4.3 Blockchain technology diffusion of innovation in tourism

#### 4.3.1 Relative advantage

Relative advantage refers to the degree to which blockchain is perceived as better than other technologies. Perceived benefits are linked to the perceived strategic value and the perceived usefulness of the technology [69]. While social influence has some impact on perceived benefits, organizations nevertheless need to see the benefits for themselves. Potential users will only adopt a technology if it confers economic or functional benefits [70]. Thus, the greater the perceived benefits of blockchain are, the higher the adoption rate of the technology by the tourism industry. Three respondents assumed that the perceived benefits of blockchain outweighed other elements of diffusion theory. If a company's strategic value is based on innovation or on positioning itself as innovative, it will use new technologies faster than others. For example:

*“I think is a problem of benefits. Like I mean wasn’t it complex to wire all the water with cables for electricity. Yeah, it was. It was the biggest feat of modern times. Yeah, but we did it anyway because we saw the benefit from that was fairly bigger than the effort to build. And until we see that, the travel industry doesn’t get that edge that progress from blockchain.” (Respondent 1)*

*“We haven’t seen many sales from this channel (blockchain reservation system), but we have seen publicity and being seen as innovative.” (Respondent 5)*

The statement links to the concept that a relative advantage can also be an intrinsic value such as social prestige [25,71]. When there are intrinsic relative advantages such as ‘prestige stimulate organizations,’ they will be motivated to adopt innovations [72,73].

#### 4.3.2 Compatibility

According to Rogers [25], new technologies are more likely to be adopted if they are compatible with an organization's existing systems. Our findings show that blockchain technology is compatible with companies' business operations that possess well-developed legacy systems. In the current blockchain landscape, respondents require an additional system or protocol that connects networks so that blockchain is interoperable. Interoperability is crucial so that blockchain can exchange off-chain data and transaction information, not just information within its network or system.

*“The thing is that with blockchain, in most of the cases you don't have like an out of the shelf solution. We have to develop something specific to a customer. So, it's not out of the shelf or at least not yet. It will come surely then to make that solution compatible with existing one.” (Respondent 10)*

If a technology is compatible with users’ current work processes, users will see that the technology may better execute their tasks and enhance their productivity [74]. For instance, if employees are used to a hierarchical structure or power balance and a new technology disrupts this, the adoption rate of that technology might be limited [75]. Four respondents suggested that the tourism sector first needs to address the challenge of digitalization to prepare for a digital future [76] and use technologies such as blockchain.

#### 4.3.3 Complexity

The tourism industry, intuitively, is not a high technology industry. Three respondents believed that for tourism businesses, commercialization is more important than developing technology. Since the implementation of blockchain is a backend procedure, travelers do not usually see the complexity of the technology. Instead, the users of the technology and internal customers are employees of the tourism companies. Respondents mentioned that while the technology is not difficult to learn, the ‘change’ (to systems, processes, and procedures) makes it complex. The skills and efforts required of an adopter to utilize the innovation are closely related to its complexity [77]. When users are motivated to adopt and learn about the technology, there are ways to make the execution smoother. Additionally, *“the integration was not going to be through the blockchain itself, it's going to be at the API level. So, it's going to be like any other system” (Respondent 10)*. Likewise, blockchain technology can come with a steep learning curve for many tourism enterprises, even though early adopters may not perceive it as complex.

#### 4.3.4 Trialability

Six respondents pointed out misconceptions about blockchain's trialability and required significant investment. Previous studies have stated that only large corporations can access blockchain and that blockchain's trialability is limited [78]. However, eight out of ten respondents in this study are startups, and all of them were able to try the technology before fully implementing it. The interview results show that blockchain has evolved to the point that even small enterprises such as startups can easily access and test the technology.

*“You can do some sandboxes environment, some staging environment where you can have some small groups of users that we’ll call them early adopters. […] So that can give users a bit of security and confidence with how these things work.” (Respondent 1)*

Trialability is a great way for users to observe the benefits of the technology and reduce the perceived risks of its use [79]. Through testing and trials, users can explore whether blockchain meets their business needs. Misconceptions about the new technology can be eliminated by trying it for a while [80]. In addition, trialability positively correlates with product familiarity [81], and the more familiar people are with blockchain technology, the fewer misconceptions they will have about it. All respondents in this study stated that they had tested the technology before their organizations had fully adopted it.

#### 4.3.5 Observability

Our results show a limited number of successful cases for potential users to observe, as cryptocurrencies still receive much more media focus than other technology uses. Respondents confirmed the limited observability of the technology:

*“We try to understand what the limitations are, but I think it's just that we haven't seen any like successful use cases where you see the benefits financially yet.” (Respondent 5)*

*“The industry is too much focused-on cryptocurrency [..] rather than focusing on the use cases of the blockchain. Those use cases are so valuable people are investing in those cryptocurrencies knowing that these use cases will change the industry completely.” (Respondent 8)*

*“The more developments we see in blockchain, the more people are likely to adopt this technology.” (Respondent 3)*

Observability impacts perceived benefits [79] and can reduce perceived risks and reduce misconceptions about new products [71]. Unlike physical products, the need for the observability of information in the case of innovative products is higher [82]. This is because a lack of awareness of such products is one of the main obstacles to their adoption [83]. With the continuing rise in the adoption and use of bitcoin and cryptocurrencies, some respondents believe that blockchain technology will eventually have a wider audience. At present, for tourism, major airlines such as TUI, Lufthansa, and Air France have implemented blockchain. However, actual returns on investment have not yet been widely reported. The successful diffusion of technology requires that the use of technology move beyond early adopters [84]. Even though MacVaugh and Schiavone [85] argued that the nonadoption of technology by the majority often means the failure of its diffusion, it is important to note that blockchain technology is still at an early stage of adoption and use.

**Fig. 1:**
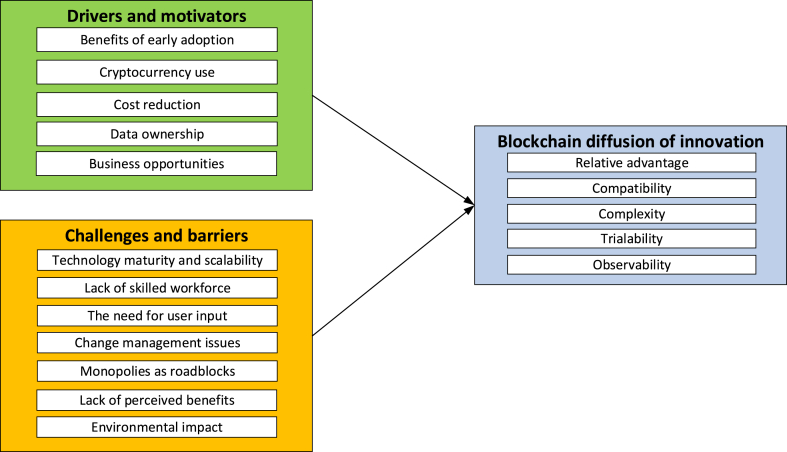
Conceptual framework of blockchain diffusion of innovation.

The conceptual framework in Fig. 1 illustrates the antecedents of blockchain diffusion of innovation.

From the results, we offer the following research propositions based on the antecedents (motivators and drivers and challenges and barriers) of blockchain diffusion of innovation for enterprises to achieve a competitive advantage (Table 4).

**Table 4:** Research propositions.

Antecedents	Propositions
Motivators and drivers of blockchain diffusion	
•Benefits to early adoption	Early adopters of blockchain in the tourism industry will gain early mover advantage (e.g., new product offering, access to suppliers, product/service differentiation, cheaper products, and publicity or prestige).
•Cryptocurrency use	The use of cryptocurrency will drive blockchain implementation.
•Cost reduction	Early adopters of blockchain in the tourism industry will benefit from lower business costs (e.g., operating, transaction and distribution costs).
•Data ownership	Blockchain adoption will enable tourism enterprises' sovereignty (e.g. eliminate dependency on intermediaries and control of their data).
•Business opportunities	Blockchain adoption opens up new business opportunities for collaboration.
Challenges and barriers to the diffusion of blockchain	
•Technology maturity and scalability	Overcoming blockchain's lack of interoperable functionality will boost its scalability.
•Lack of skilled workers	Difficulty in recruiting blockchain specialists will be a barrier to the company's blockchain development.
•The need for user input	Lack of enterprise understanding of blockchain and for organizational needs will impede its adoption Blockchain complexity is a potential impediment to applications in consumer-to-business.
•Change management issues	Resistance to change company-wide and industry-wide due to potential disruption will hinder blockchain adoption.
•Monopolies as roadblocks	Blockchain will decentralize the current travel ecosystem but private blockchains by major players will create barriers to entry for new enterprises.
•Lack of perceived benefits	Understanding of economic benefits such as use cases in the tourism context will enable blockchain adoption.
•Environmental impact	Incorporating sustainability strategy and overcoming transaction inefficiencies will allay media misconception about blockchain's energy consumption.

## 5 Theoretical and practical implications

This study serves an exploratory purpose and contributes to addressing the research gap in empirical blockchain studies. It explains how blockchain is currently used in tourism and the possible challenges facing its wider diffusion in the industry. The challenges hindering blockchain diffusion are addressed; therefore, practitioners can make decisions based on the research findings. Future researchers can use the perspectives of early adopters captured by this study as a conceptual foundation for developing hypotheses.

The empirical findings provide a holistic understanding of blockchain's opportunity, including the current and potential usages of the technology. How the respondents applied blockchain can also inspire tourism practitioners to learn to benchmark and apply it in their business operations. Enterprises can see innovative ways of using blockchain, based on the few use cases in this study. Tourism practitioners can understand the current state of blockchain technology implementation. Governments can also understand the need to intervene in the blockchain sector and use it as an area to regulate policies [86]. Therefore, governments and decision-makers can discern ideas for their future regulations. Blockchain service providers can see the drawbacks of the diffusion of new technology and use this information to implement improvements.

This study highlights the importance of the observability attribute and customer readiness. To accelerate customer adoption of new technology, marketers can enhance information reach [82]. The observability of blockchain use cases is still limited. Therefore, this academic study also contributes to this attribute by serving as a reference. Moreover, the findings show that many businesses have not yet adopted blockchain, as they do not understand the benefits of the technology. It is a significant finding, as marketers can now adjust how they promote their blockchain services. Customer readiness seems to be a major challenge; therefore, educating potential adopters should be a way to enhance their perceived benefits [10]. Accordingly, marketing practitioners can explore the challenges and barriers of the diffusion of blockchain and use this information to improve and to accordingly find innovative marketing pursuits.

Enterprises can discern ways to reduce costs and accelerate business operations. For potential blockchain users, the study illustrates the advantages and disadvantages of blockchain. They can assess whether the implementation of this cutting-edge technology is in line with their strategic goals. Compatibility has been widely discussed throughout this study. It is not only technical compatibility that must be considered but also whether the technology is compatible with organizational goals. Firms can therefore assess before they decide to commit to this technology fully. They can also do trials to see if it fits their needs. This paper enhances knowledge at the firm level by providing critical analysis on blockchain diffusion of innovation. It is pertinent to the literature to note that users will replace new technology only if it is well matched to their goals [74].

Blockchain addresses many challenges, but as Peck [87] argued, blockchain might not be necessary if a traditional database still fits organizational needs and a fast transaction speed is not needed. Moreover, if a project does not require a database and no data are to be recorded, then there is no need to use an advanced system. As a result, as Felin and Lakhani [88] postulated, companies must be able to provide satisfactory answers to the following three questions before adopting blockchain: 1) what are the company's short- and long-term goals, 2) what are blockchain's capabilities, and 3) what problems are they seeking to solve with this technology? These questions are necessary to elicit the readiness for successful blockchain adoption, ensure seamless implementation and reap the expected benefits.

### 5.1 Summary of examples of use cases in tourism

#### 5.1.1 Distribution and reservation system

Blockchain can enable direct and peer-to-peer transactions between hotels and travelers, eliminating the need for intermediaries and reducing costs. It can help automate booking processes, reducing the risk of double bookings. Additionally, the immutability of blockchain records enhances trust and accountability, as all transactional data are stored in a tamper-proof manner, preventing fraud and enhancing the overall booking experience for both hotels and guests. Example business use cases are Winding Tree and Nordic Choice Hotels [12,13].

#### 5.1.2 Payment service

One of the original use cases of blockchain rooted in cryptocurrencies is payment processing by providing secure, transparent, and efficient transactions. With blockchain, payments can be executed directly between parties, eliminating the need for intermediaries (e.g., banks) and reducing transaction costs. An example business use case is Travala's incorporation of cryptocurrencies [50].

#### 5.1.3 Loyalty programs

By integrating blockchain into loyalty programs, companies and customers can eliminate potential disputes over loyalty points. Blockchain-driven loyalty programs are verifiable and secured. For example, Singapore airline's Krispay loyalty program converts flying miles into cryptocurrency and securely stores them in customers' digital wallets [8,89].

#### 5.1.4 Identity management

Blockchain enables people to selectively share their identity documents with trusted parties and minimize the risk of data breaches. Decentralized data platforms where users have full control over their data can be beneficial [53].

#### 5.1.5 Smart contracts

With blockchain-driven smart contracts, the auditing and reporting capabilities of organizations can be improved, which will eventually enhance the service quality of organizations [10]. For example, TUI uses the smart contract concept and private blockchain to manage their room inventories [90].

#### 5.1.6 Air cargo tracking

Real-time air cargo tracking and baggage tracing can be enhanced using blockchain technology. Utilizing an immutable ledger, each step of the baggage's route can be recorded, such as the origin, destination, and intermediate stops. This use case is proven to be more than theoretical by a Danish company called Blockshipping [91].

## 6 Conclusion

Based on the results of this study, blockchain technology contributes to the tourism ecosystem by removing intermediaries, thus reducing costs and accelerating operational processes. With the help of this technology, businesses can access new markets and business opportunities. Contrary to claims made by the media, which tended to suggest that “blockchain is complex,” “difficult to understand,” and “expensive,” most respondents seem to believe that these are not significant factors influencing the diffusion of blockchain in the tourism industry. Their assertions and explanations of why they think blockchain diffusion has been limited in tourism can be explained by Rogers' [23] diffusion of innovation theory. Their assumptions on the slow diffusion rate of blockchain in tourism are contingent on blockchain technology's relative advantage, compatibility, and observability attributes. The early adopters and innovators interviewed stated that they have seen more noneconomic benefits, such as prestige, publicity, and innovativeness, than actual financial benefits. Therefore, improving blockchain's perceived benefits and relative advantage can surely accelerate blockchain diffusion.

### 6.1 Limitations

This study has covered an ambitious context of the tourism ecosystem. While this can express an optimistic prediction of uncovering nuances and linkages in blockchain studies in tourism, it also embraces some limitations. Since awareness about blockchain is very limited in the tourism sector, it was challenging to identify and recruit early adopters and innovators who possess profound blockchain knowledge and the travel industry. Additionally, it is important to note that the study focuses on early adopters beyond cryptocurrencies; therefore, the sample size used in this research is limited. It can be influenced by the biases of the individuals who created the data that were used in this research. We conducted semistructured interviews during the COVID-19 pandemic. Investment in blockchain during this period could have been adversely affected, as the tourism sector suffered immense losses due to lockdowns and travel restrictions. Our propositions offer a research agenda for the strategic implementation of blockchain and are not limited to the tourism sector. Despite these limitations, this research provides valuable insights into the topic under investigation and can serve as a foundation for future studies in this field.

### 6.2 Future research

We also encourage future empirical studies to consider testing our propositions via surveys of the perceptions and awareness of users and enterprises with larger sample sizes and beyond the tourism sector. Future studies can also investigate the decentralization effect of blockchain on the existing power structure of monopolies in any specific sector or industry.

Additionally, studying the impact of blockchain on enhancing trust, transparency, and security in various aspects of tourism, such as reservation systems and identity verification, will contribute to building a robust and reliable tourism ecosystem. Given the current discourse surrounding the environmental impact of tourism, it is pertinent to explore blockchain solutions that consume lower levels of energy. Such studies can provide valuable insights into the development of energy-efficient blockchain frameworks. Considering the socioeconomic implications and potential barriers to adoption, it is also beneficial to explore the correlation between regulatory frameworks and user acceptance of blockchain technology for sustainable growth and positive industry transformation in the tourism sector.

## Data availability statement

Data included in article/appendix/referenced in article

## CRediT authorship contribution statement

**Yin Maythu:** Writing – review & editing, Writing – original draft, Visualization, Validation, Software, Resources, Project administration, Methodology, Investigation, Formal analysis, Data curation, Conceptualization. **Andrei O.J. Kwok:** Writing – review & editing, Writing – original draft, Visualization, Validation, Supervision, Methodology, Formal analysis, Conceptualization. **Pei-Lee Teh:** Writing – review & editing, Visualization, Validation.

## Declaration of competing interest

The authors declare that they have no known competing financial interests or personal relationships that could have appeared to influence the work reported in this paper.

## Footnotes

1 Name of certificate is anonymized.
