# Supplementary material for: Blockchain technology diffusion in tourism: Evidence from early enterprise adopters and innovators
Source: Heliyon. 2024 Jan 14;10(2):e24675. doi: 10.1016/j.heliyon.2024.e24675 (PMC10833103; doi:10.1016/j.heliyon.2024.e24675)
Supplement: Multimedia component 1 [file mmc1.docx]

**Appendix**

Authors’ illustration of the coding phases leading to the theme formation.

**1. Initial coding stage**

**2. Clustering codes by relevance**
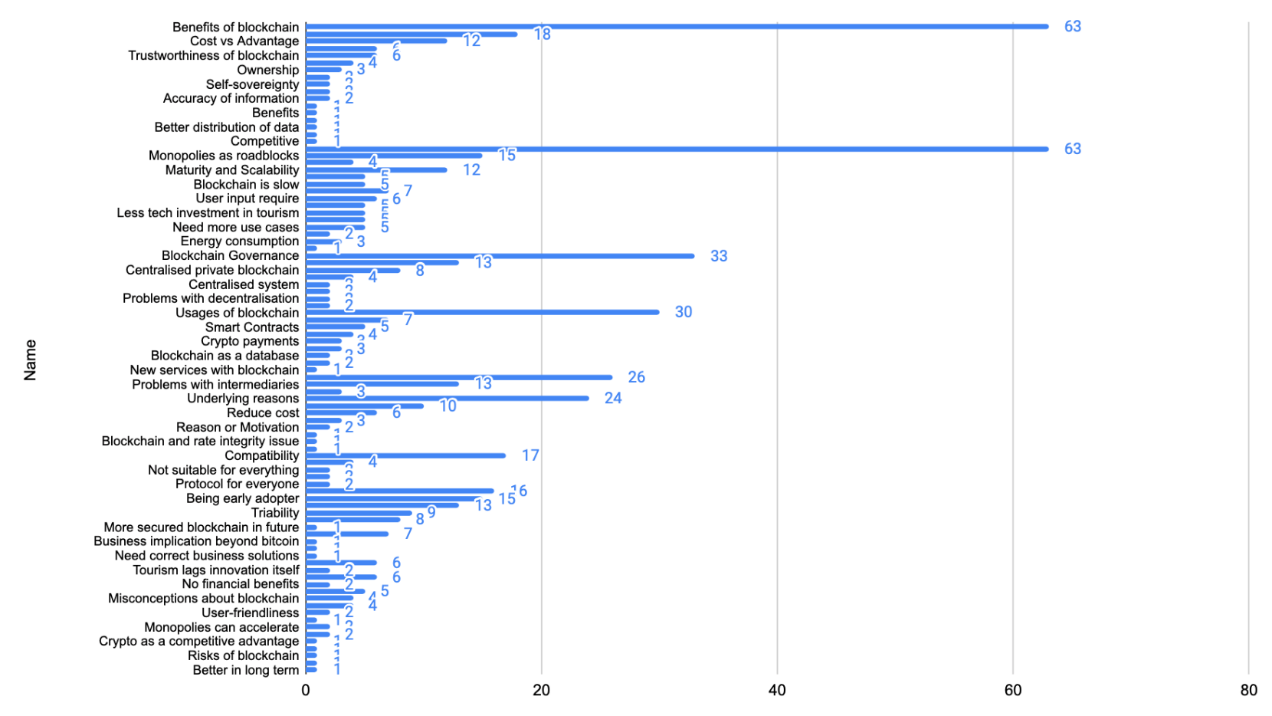


**3. Theme formation**

| Benefits of blockchain | Challenges | Diffusion of blockchain |
| --- | --- | --- |
| Efficiency | Monopolies as roadblocks | Loyalty program |
| Cost vs Advantage | Market barriers | Smart Contracts |
| Blockchain is secure | Maturity and Scalability | Reviews |
| Trustworthiness of blockchain | Blockchain tradeoff | Crypto payments |
| Transparency | Blockchain is slow | Blockchain for community |
| Ownership | Change Management Issues | Blockchain as a database |
| Immutability | User input require | Reaching to different suppliers |
| Self-sovereignty | Lack of human resources | New services with blockchain |
| No fraudulence | Less tech investment in tourism | Blockchain Governance |
| Accuracy of information | No real solution | Government regulation |
| Traceability | Need more use cases | Centralised private blockchain |
| Benefits | Environmental impact | Government initiatives |
| Blockchain makes easier communication | Energy consumption | Centralised system |
| Better distribution of data | Problems with intermediaries | Government regulation - crypto |
| Consistency in protocols | Problem with centralisation | Problems with decentralisation |
| Competitive | Compatibility | Controling a blockchain network |
| Removing intermediaries | Interoperability |  |
| Reduce cost | Not suitable for everything |  |
| Using crypto in travel | Digital and compatible |  |
| Reason or Motivation | Protocol for everyone |  |
| Blockchain and integrity |  |  |
| Blockchain and rate integrity issue |  |  |
| Blockchain for analytics |  |  |
